# Predictors of Referral to Cardiac Rehabilitation in Patients following Hospitalisation with Heart Failure: A Multivariate Regression Analysis

**DOI:** 10.3390/jcm11051232

**Published:** 2022-02-24

**Authors:** Catherine Giuliano, Don Vicendese, Sara Vogrin, Rebecca Lane, Andrea Driscoll, Diem Dinh, Katie Palmer, Itamar Levinger, Christopher Neil

**Affiliations:** 1 Institute for Health and Sport, Victoria University, Melbourne, VIC 8001, Australia; rebecca.lane@vu.edu.au (R.L.); itamar.levinger@vu.edu.au (I.L.); christopher.neil@unimelb.edu.au (C.N.); 2 Department of Cardiology, Western Health, Sunshine Hospital, Melbourne, VIC 3021, Australia; 3 Melbourne School of Population and Global Health, University of Melbourne, Melbourne, VIC 3010, Australia; don.vicendese@unimelb.edu.au; 4 Department of Mathematics and Statistics, La Trobe University, Melbourne, VIC 3086, Australia; 5 Australian Institute for Musculoskeletal Science (AIMSS), University of Melbourne and Western Health, St Albans, VIC 3021, Australia; sara.vogrin@unimelb.org.au; 6 College of Health and Biomedicine, Victoria University, Melbourne, VIC 8001, Australia; 7 Centre for Quality and Patient Safety, School of Nursing and Midwifery, Deakin University, Geelong, VIC 3216, Australia; andrea.driscoll@deakin.edu.au; 8 Department of Cardiology, Austin Health, Melbourne, VIC 3084, Australia; 9 Centre of Cardiovascular Research and Education in Therapeutics, School of Public Health and Preventive Medicine, Monash University, Melbourne, VIC 3004, Australia; diem.dinh@monash.edu; 10 Department of Physiotherapy, Monash Health, Melbourne, VIC 3175, Australia; katie.palmer@monashhealth.org; 11 School of Primary and Allied Health Care, Monash University, Frankston, VIC 3199, Australia; 12 Department of Medicine, Western Health, The University of Melbourne, Melbourne, VIC 3010, Australia

**Keywords:** heart failure, referral, cardiac rehabilitation, exercise training

## Abstract

Background: This exploratory observational case–control study investigated the rate of referral to cardiac rehabilitation (CR) among patients hospitalised with heart failure (HF) and identified factors associated with referral. Methods: Patients hospitalised with HF as identified by the Victorian Cardiac Outcomes Registry HF study were included. Factors found to be univariately associated with referral were selected for multivariate logistic regression. Results: Among 1281 patients (mean age: 76.9 years; 32.8% HFrEF and 33.9% HfpEF), 125 (9.8%) were referred to CR. Patients referred were younger (73.6 (2.7, 81.5) vs. 80.2 (71.1, 86.5) *p* < 0.001) and were more likely to be men (72%, *p* < 0.001). Factors associated with referral included inpatient percutaneous coronary intervention (OR, 3.31; 95% CI, 1.04–10.48; *p* = 0.04), an aetiology of ischaemic or rhythm-related cardiomyopathy, and anticoagulants prescribed on discharge. Factors that lowered the likelihood of referral included older age, female, receiving inpatient oxygen therapy, and the presence of chronic obstructive pulmonary disease (COPD) or anaemia. Conclusions: The rate of referral to CR following hospitalisation with HF is low. Shortfalls are particularly evident among females, older patients, and in those with COPD or anaemia. Future studies should focus on improving referral processes and translating proven strategies that increase referrals to CR into practice.

## 1. Introduction

Heart failure (HF) is a complex syndrome that affects more than 30 million people worldwide [1,2] and results in significant clinical, functional, and financial costs to individuals and the community [1,3]. The prevalence of HF is expected to grow due to the global aging population, increasing the prevalence of HF risk factors and improving post-myocardial infarction survival [4]. Symptomatically, patients with HF experience a significant burden including exercise intolerance, dyspnoea, and fatigue [5].

In Australia, cardiac rehabilitation (CR) offers a supervised program of clinical exercise prescription, education, and risk factor modification to patients with cardiovascular disease. CR programs are integrated across acute (phase I), outpatient (phase II), and community-based settings (phase III), and referral is often made following an acute cardiovascular event. CR is an integral component in the treatment paradigm for HF with evidence consistently demonstrating the reversal of muscle dysfunction and increased aerobic capacity following CR [6,7,8]. Suitably designed exercise programs may also reduce premature mortality and the risk of hospitalisations, as well as improve quality of life, regardless of disease severity [9,10]. Despite these benefits, CR programs may be underutilised in Australia, with only 30–50% of eligible patients engaging in phase II CR [11,12,13]. These data, however, mostly represent patients with acute coronary syndrome or patients undergoing coronary revascularisation procedures [11,12,13]. Importantly, patients with HF are clinically distinct from individuals with other cardiovascular conditions; they are uniquely characterised by comorbidity and older age, while the progressive and deteriorating nature of the syndrome is underpinned by recurrent exacerbations and a gradual decline in functional capacity. As such, patients with HF should be uniquely considered in investigations of CR engagement.

One previous study from the USA investigated rates of referral to CR among 105,619 patients hospitalised with HF [14] and found that only 10% of eligible patients received CR referral at the time of discharge. Multivariate analysis showed that younger age, fewer comorbid conditions, and in-hospital procedures (i.e., coronary artery bypass grafting, percutaneous coronary intervention, and cardiac valve surgery) were most strongly associated with CR referral. These findings offer some important considerations for enhancing targeted CR engagement; however, due to the possible influence of patient insurance and program eligibility criteria, these findings may not be transferrable to the other nations, including Australia. The current utilisation of CR among patients with HF in Australia remains largely unknown, as do the factors that influence referral and participation in this patient group. This study investigated the rates of referral to CR among patients following hospitalisation with HF in Victoria and identified factors associated with referral and participation.

## 2. Materials and Methods

This exploratory observational case–control study is nested within the Victorian Cardiac Outcomes Registry Heart Failure study (VCOR-HF) [15]—a prospective longitudinal cohort study involving patients admitted to Victorian Hospitals with an acute episode of HF. The VCOR-HF study (described in detail elsewhere [15]) was rolled out across 16 hospital sites and enrolled all adults hospitalised with a primary diagnosis of HF over a 1 month period annually, between the years of 2014 and 2017. Data collected included patient demographics, medical history, cardiac risk factors, HF aetiology, clinical measures, cardiac investigations (i.e., echocardiogram and angiogram), inpatient procedures received, discharge medications, discharge clinical status, and referral information. The primary outcome, referral to CR, included patients who were referred to either outpatient cardiac rehabilitation or a specific HF exercise program. An analysis of actual attendance was completed at 30 days post discharge. Attendance was defined as a patient attending at least one appointment to cardiac rehabilitation.

The research protocol was approved by Ethics Committees from Melbourne Health and Victoria University, as well as the VCOR Data Research and Publications Committee.

Patients included in this study were identified from the VCOR-HF study. Patients were only excluded from this study if they were discharged to palliative care or died in hospital. The study population was then grouped according to the status of referral to CR on discharge (i.e., referred to CR or not referred).

### 2.1. Statistical Analysis 

Descriptive statistics are presented as the median and interquartile range (IQR; [25th percentile, 75th percentile]) for continuous variables (none of the data were normally distributed) and frequency and percentage for categorical variables. Baseline patient characteristics were compared between CR referral groups using Mann–Whitney nonparametric tests for continuous variables and Pearson’s chi-square or Fisher’s exact test for categorical values. The unadjusted effect of each factor of interest on referral to CR was evaluated using univariate logistic regression and presented as odds ratios (ORs) and 95% confidence intervals (CIs). Factors found to be univariately associated with CR referral were selected for multivariate logistic regression to assess the independent effect of each factor on the outcome, while adjusting for all other factors of interest. The covariates of age and gender were included in the multivariate logistic regression, regardless of univariate associations. Multivariable models were developed through backward and forward elimination methods [16] using SPSS automatic algorithms [17]. The model fit (i.e., its capacity to distinguish cases and controls) was evaluated using likelihood ratio tests (LRTs), Akaike information criterion (AIC) [18], and a calculation of the area under the receiver operator curve (AUC); the best-fitted model was selected as the final model.

The influence on model selection from several variables that had a high proportion of either missing observations or categories defined as unknown was assessed by a sensitivity analysis, by separately building models that included and then excluded these variables. All statistical analyses were performed using SPSS software, version 23.

### 2.2. Subgroup Analysis

Univariate and multivariate modelling was repeated for subgroups of patients with HF with reduced ejection fraction (HFrEF) (EF ≤ 40%) and HF with preserved ejection fraction (HFpEF) (EF ≥ 50%) [8].

## 3. Results

### 3.1. Study Flow and Referral to CR

**Figure 1:**
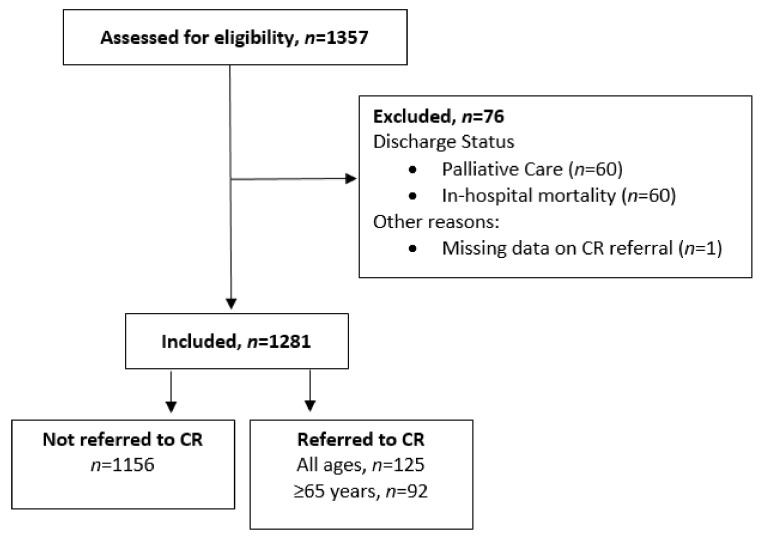
Study flow and referral to Cardiac Rehabilitation (CR).

Study flow and referral outcomes are shown in Figure 1. A total of 1357 patients were identified in the index dataset. Of these, 76 patients were excluded due to hospital discharge to palliative care (*n* = 15), in-hospital mortality (*n* = 60), or missing CR referral data (*n* = 1), leaving 1281 patients who were potentially eligible for referral to CR at time of discharge (median age: 76.9 years (70.3, 86.3); 32.8% with HFrEF and 33.9% with HFpEF). At the time of discharge, 125 (9.8%) patients were referred to CR (median age: 73.6 years (62.7, 81.5); 28% females). This included 92/1087 (8.5%) adults ≥ 65 years, 62/420 (14.8%) patients with HFrEF, and 26/434 (6%) patients with HFpEF.

### 3.2. Baseline Characteristics of the Population

Select baseline characteristics of the population are presented in Table 1. A complete baseline characteristics table is reported in the Supplementary Materials. Patients referred to CR were younger (73.6 [62.7, 81.5] vs. 80.2 years [71.1, 86.5], *p* < 0.001) and were more likely to be men (72% vs. 28%, *p* < 0.001). In patients where the HF subtype was known, there was a statistically significant difference for level of HF subtype (*p* < 0.001), with the main difference being that referred patients were more likely to have HFrEF than HFpEF (49.6% vs. 20.8%) compared to non-referred patients (30.1% vs. 35.3%). The presence of hypertension, dementia, chronic obstructive pulmonary disease (COPD)/asthma, chronic kidney disease, iron deficiency, and anaemia was significantly more frequent in the non-referred group compared to the referred group, whereas the proportion of patients with ischaemic or arrhythmia-related HF aetiology was significantly greater in the referred group compared to the non-referred group (*p* = 0.001 and 0.04, respectively). Patients not referred to CR had a significantly greater number of medications prescribed on discharge (9 (8, 11) vs. 10 (8, 13), *p* = 0.047).

### 3.3. Factors Associated with Referral to CR

Factors associated with referral to CR at the time of discharge are presented in Table 2, including inpatient percutaneous coronary intervention (PCI) procedure (OR, 3.31; 95% CI, 1.04–10.48; *p* = 0.04), an aetiology of ischaemic-related or rhythm-related cardiomyopathy, and anticoagulants prescribed on discharge. Factors that lowered the chance of referral included older age, female, receiving inpatient oxygen therapy, and the presence of COPD or anaemia.

### 3.4. Sensitivity Analysis

Due to missing data, the variables of smoking history, HF subtype, and estimated ejection fraction were excluded from the model; the sensitivity analysis resulted in identical final variables found to be independently associated with the outcome; thus, these variables were included in the final models.

### 3.5. Subgroup Analysis

Factors associated with referral to CR by multivariate analysis by HF subgroup type are shown in Table 3. For patients with HFrEF, factors significantly associated with increased referral to CR included inpatient PCI (OR, 4.91; 95% CI, 1.27–18.92; *p* = 0.02), discharge heart rate, and implantable cardiac defibrillator inserted during hospital admission (refer to Table 3 for ORs, CIs, and *p*-values), while the prescription of antiplatelets on discharge was significantly associated with decreased odds of referral. No association was found for age among patients with HFrEF. For patients with HFpEF, factors that significantly increased the odds of referral included ischaemic or rhythm-related aetiology, while receiving inpatient oxygen therapy significantly decreased odds of referral. No associations were found for age and gender among patients with HFpEF.

### 3.6. Attendance at 30 Days Post Discharge 

At 30 days post discharge, 55 patients had attended at least one cardiac rehabilitation appointment, while the remaining were either still pending or had missing follow-up data.

## 4. Discussion

### 4.1. Key Findings

**Figure 2:**
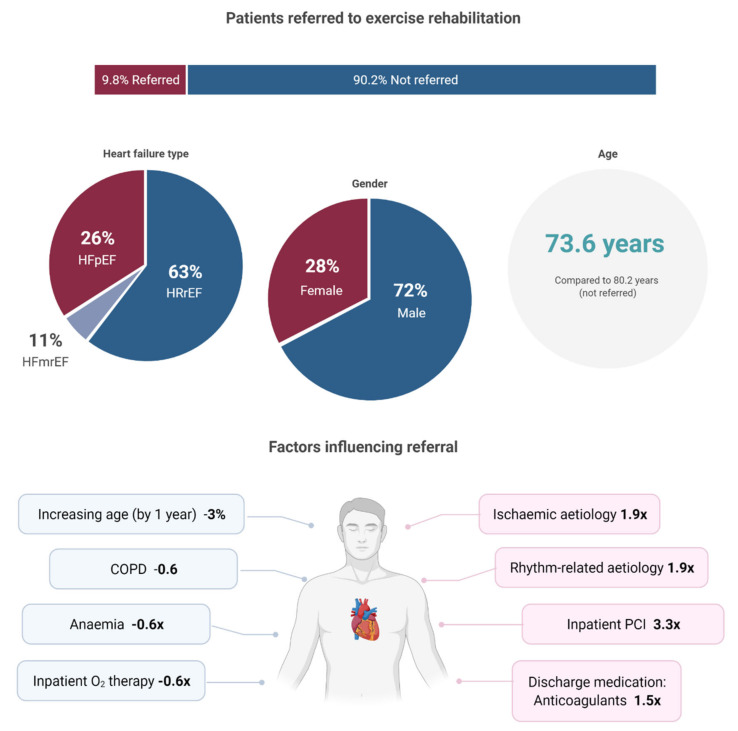
Central illustration of key findings (created with BioRender.com). Bottom image: Factors which decreased the odds of referral are shown in blue, while factors that increased the odds of referral are shown in pink. The multiplication factor is derived from the odds ratios reported in Table 2, Section 3.3. HFpEF; Heart failure with preserved ejection fraction, HFrEF; Heart failure with reserved ejection fraction, HRmrEF; Heart failure with mid-range ejection fraction, COPD; Chronic obstructive pulmonary disease, PCI; Percutaneous coronary intervention.

This exploratory study provides unique insight into CR referral practices among patients hospitalised with HF. The key findings are summarised in Figure 2. In the study population, <10% of patients were referred to CR following hospitalisation with HF. On multivariate analysis, younger age, male, receiving an inpatient PCI, an aetiology of ischaemic or rhythm-related cardiomyopathy, and receiving anticoagulants on discharge were most strongly associated with CR referral. Factors that were most strongly associated with non-referral included older age, female, receiving oxygen therapy, and the presence of COPD or anaemia.

### 4.2. Rates of Referral to CR

Clinical practice guidelines recommend that all patients with HF should be referred to a CR program. To our knowledge, this is the first Australian study to report CR referral data specifically among patients hospitalised with HF. We report that only one-tenth of the study population received a referral to CR on discharge. While international data in this population are also sparce [14], the rate of referral reported in the current study is consistent with a study from the USA in the same patient population [14], despite the potential differences in program funding and eligibility between the two nations. Moreover, both studies found that the proportion of referral was higher among patients with HFrEF than those with HfpEF. Importantly, we only identified patients who were referred to CR (i.e., the first step to program engagement); hence, actual participation is likely to be lower still.

The issue of poor engagement in CR is not unique to patients with HF, although the issue appears to be more significant in this population. In a large prospective audit of 2299 patients hospitalised with acute coronary syndromes across Australia and New Zealand, only 46% were referred to CR on discharge [19]. Another Australian study found a greater proportion of referrals to CR—by two-thirds—in patients with cardiovascular conditions other than chronic HF [12]. This emerging pattern is perhaps not surprising, given the evidence supporting CR for patients with HF lagged that for other cardiovascular conditions by as many as 14 years [20,21]. It is plausible that cultural differences in the considered importance of CR for patients with acute ischemic conditions compared to those with HF may influence clinicians referring practices. Suitably designed future studies should consider investigating this hypothesis.

Several strategies have been suggested to improve the CR referral process, such as electronic referral systems [22], integrating referral to CR into the quality assessment of HF management [23,24], and pre-printed hospital discharge orders [23]. Optimising opportunities to facilitate continuity of care such as having a dedicated and consistent team of health professions and established pathways of communication between inpatient and outpatient CR facilitators has also been recommended [25]. Despite these strategies, our data suggest that low referral rates remain a key issue affecting engagement in CR among patients with HF, and further work is required to translate these previously proven strategies into practice.

### 4.3. Factors Associated with Referral and Non-Referral

This study identified several factors that significantly influenced the odds of referral and non-referral. Notably, for every year increase in age, the odds of referral decreased by 2% (95% CI: 1%, 4%), while the presence of COPD or anaemia reduced the likelihood of referral by 41% (95% CI: 1%, 64%) and 48% (95% CI: 13%, 69%), respectively. It is well established that older individuals are disproportionately affected by HF [26]. Older patients with HF are characterised by a higher incidence of comorbidities such as COPD, anaemia, sarcopenia, and frailty [26,27,28,29,30], and they experience higher rates of hospitalisation and clinically adverse events in comparison to younger individuals with HF [27,31]. These factors present a challenging paradox, as patients with more complex presentations or patients who are older may have the greatest need for CR. While engaging older adults in CR is challenging, it is possible, and significant benefits can be achieved with exercise training [32,33,34,35,36]. In this regard, older individuals represent a group of patients that may benefit the most from engagement with CR, and referring these individuals should be considered a priority.

Patients with HFrEF were more likely to be referred compared to patients with HFpEF (63% vs. 26%). It is known that individuals with HFpEF are generally older than patients with HFrEF and are more likely to be female. It is also plausible that the difference in referrals between HF subtypes may be due to a relatively smaller evidence base supporting exercise training for patients with HFpEF [37], and there may be a lag in translation to practice for this patient group. This hypothesis may also apply to our finding that patients who received an inpatient PCI and were 3.3 times more likely to receive a referral, while the odds of referral almost doubled in patients with an ischaemic or rhythm-related aetiology.

Gender is an important clinical consideration across all specialities. Although not found to be statistically significant in this study, females had half the odds of referral to CR among all patients and in subgroups of HFpEF and HFrEF.

### 4.4. Limitations

This study had some potential limitations. First, this analysis determined eligibility for CR as any patient who was discharged to a home residence. In reality, not all patients discharged home may be eligible for CR due to contraindications to exercise training, for instance, due to severe aortic stenosis or uncontrolled diabetes [38]. Second, this study assessed referral status on the basis of a single hospital admission, and we were unable to ascertain whether patients had already attended CR following a previous hospital discharge. Likewise, the data did not capture referrals that may have been initiated by the primary care physician following hospital discharge. Third, this study only assessed the influence of a select number of factors on CR referral. It did not consider possible individual patient-, clinician-, or system-related influences on referral and participation, including the proportion of patients who were offered referral to CR but declined. Further research investigating a possible cultural influence among referring practitioners and patients is important. Lastly, whilst the modelling completed was suitable for the question and the data available at this time, we acknowledge that results may be biased due to the small sample size. Despite these limitations, this is the first study to evaluate factors influencing referral to CR in an Australian context and, as such, provides valuable insight into the current issues facing rehabilitation engagement in the HF population.

## 5. Conclusions

This study highlights a variation in CR referral practices across patient characteristics in those hospitalised with HF. Proportionately more men than women receive a CR referral, while younger patients and those who received an inpatient PCI are more likely to receive a CR referral. These findings highlight the ongoing issue of poor referral and engagement in CR in patients with HF and the persistent disparity between guideline recommendations and actual clinical practice. Further work is required to translate previously proven effective strategies to increase CR referrals into practice.

## Figures and Tables

**Table 1:** Select baseline patient characteristics among all patients and those referred and not referred to cardiac rehabilitation.

Characteristics	All (%) (*n* = 1281)	CR Referral (%) (*n* = 125)	No CR Referral (%) (*n* = 1156)	*p*-Value
Age, years	79.7 (70.3, 86.3)	73.6 (62.7, 81.5)	80.2 (71.1, 86.5)	<0.001
Male, *n* (%)	723 (56.4)	90 (72.0)	633 (54.8)	<0.001
ǂ BMI, kg/m^2^	29.1 (35.8, 34.5)	29.7 (25.8, 34.9)	29.0 (24.8, 34.5)	
HF Subtype, *n* (%)				
HFrEF	420 (32.8)	62 (49.6)	358 (31.0)	<0.001 †
HFmrEF	169 (13.2)	11 (8.8)	158 (13.7)	
HFpEF	434 (33.9)	26 (20.8)	408 (35.3)	
Unknown	258 (20.1)	26 (20.8)	232 (20.1)	
ǂ LVEF (%)	38.0 (25.6, 50.3)	30.0 (22.3, 39.8)	40 (26.0, 53.0)	0.004
NYHA on Discharge, *n* (%)				
Class I/II	20 (4.5)/236 (53.5)	2 (0.5)/37 (68.8)	18 (4.7)/198 (51.4)	
Class III/IV	162 (36.7)/23 (5.2)	14 (25.9)/1 (1.9)	148 (38.2)/22 (5.7)	
Unknown	840 (65.6)	71 (56.8)	770 (66.7)	
Admission Speciality, *n* (%)				0.001 †
HF unit	126 (9.8)	18 (14.5)	108 (9.3)	
Cardiology	434 (33.9)	58 (46.8)	376 (32.5)	
Gerontology	36 (2.8)	5 (4.0)	31 (2.7)	
General Medicine	622 (48.6)	39 (31.5)	583 (50.4)	
Other	62 (4.8)	4 (3.2)	58 (5.0)	
Cardiovascular History, *n* (%)				
History of heart failure	968 (75.5)	93 (74)	874 (75.6)	
Previous hospitalisation for HF	774 (60.4)	75 (60.0)	698 (60.4)	
Cerebrovascular disease	242 (18.9)	27 (21.6)	215 (18.6)	
Hypertension	977 (76.2)	85 (68.0)	891 (77.1)	0.02
History of angina	481 (37.5)	43 (34.4)	437 (37.8)	
History of PCI or CABG	393 (30.7)	37 (29.6)	356 (30.8)	
History of MI	394 (30.7)	40 (32)	353 (30.5)	
Arrhythmia	695 (54.2)	66 (52.8)	628 (54.3)	
CIED therapy	284 (22.2)	29 (23.2)	255 (22.1)	0.004
ǂ Smoking status				
Current smoker	133 (12.4)	18 (16.5)	115 (11.9)	
Ex-smoker	504 (46.8)	51 (46.8)	451 (46.8)	
Heart Failure Aetiology, *n* (%)				
Ischaemic	458 (35.8)	62 (49.6)	396 (34.3)	0.001
Hypertension	223 (17.4)	16 (12.8)	207 (17.9)	
Valvular	179 (14.0)	12 (9.6)	137 (14.4)	
Arrhythmia	187 (14.6)	26 (20.8)	161 (13.9)	0.04
Other Medical History, *n* (%)				
Diabetes	552 (43.1)	56 (44.8)	496 (42.9)	
Dementia	100 (7.8)	4 (3.2)	96 (8.3)	0.04
Depression	251 (19.6)	20 (16.0)	231 (20.0)	
COPD/asthma	394 (30.8)	23 (18.4)	371 (32.1)	0.002
Obstructive sleep apnoea	187 (14.6)	17 (13.6)	170 (14.7)	
Chronic kidney disease				0.002
Mild	241 (18.8)	22 (17.6)	217 (19.0)	
Moderate	408 (31.9)	28 (22.4)	380 (32.9)	
Severe	159 (12.4)	10 (8.0)	149 (12.9)	
Iron deficiency	253 (20)	16 (12.9)	237 (20.8)	0.04
Anaemia	394 (30.8)	23 (18.4)	371 (32.1)	0.002
Treatments during Admission, *n* (%)				
IV diuretics	1096 (85.8)	102 (82.9)	994 (86.1)	
Oral diuretics	1161 (90.9)	108 (88.5)	1053 (91.2)	
Oxygen therapy	837 (65.6)	65 (53.3)	772 (66.9)	0.003
CPAP/BiPAP	174 (13.6)	13 (10.6)	161 (14.0)	
Angiography	112 (8.8)	19 (15.3)	93 (8.1)	0.01
PCI	15 (1.2)	5 (4.0)	10 (0.9)	0.002
CIED therapy				0.002
Pacemaker	28 (2.2)	3 (2.4)	25 (2.2)	
CRT-P	2 (0.2)	0 (0.0)	2 (0.2)	
ICD	15 (1.2)	5 (4.1)	10 (0.9)	
CRT-D	18 (1.4)	13 (1.1)	5 (4.1)	
Resting Haemodynamics on Discharge				
Systolic BP (mmHg)	120 (110.0, 135)	118 (110, 130)	120 (110, 135)	
Diastolic BP (mmHg)	68 (60, 75)	68 (60, 75)	68 (60, 75)	
HR (bpm)	74.0 (65, 83)	75.0 (65, 85)	74.0 (65, 82)	
HF Pharmacotherapy, *n* (%)				
ACE inhibitor	534 (41.8)	54 (43.2)	480 (41.6)	
ARB	219 (17.1)	24 (19.4)	195 (16.9)	
Beta blocker	910 (71.2)	98 (79.0)	812 (70.4)	0.04
Aldosterone antagonist	474 (37.1)	48 (38.7)	426 (36.9)	
Digitalis	210 (16.4)	15 (12.1)	195 (16.9)	
Loop diuretic	1207 (94.2)	118 (94.4)	1089 (94.2)	
Antiplatelet	674 (52.7)	60 (48.0)	614 (53.2)	
Anticoagulant	586 (45.8)	69 (55.6)	517 (44.8)	0.02
Calcium channel antagonist	209 (16.4)	21 (16.9)	188 (16.3)	
Total number of meds	10.0 (8.0, 13)	9 (8, 11)	10 (8, 13)	0.047

Data are expressed as medians and percentiles [75%, 25%] for continuous variables and counts and proportions (%) for categorical variables. ǂ Variables where missing data >15%. † Where the factor has more than one level, the *p*-value applies to the overall association of this factor with the outcome. BMI, body mass index; LVEF, left-ventricular ejection fraction; HFrEF, heart failure with reduced ejection fraction; HFmrEF, heart failure with mid-range ejection fraction; HFpEF, heart failure with preserved ejection fraction; HF, heart failure; PCI, percutaneous coronary intervention; CABG, coronary artery bypass graft; MI, myocardial infarction; CIED, cardiac implantable electronic device; CRT-p, cardiac resynchronisation therapy—pacemaker; ICD, implantable cardioverter defibrillator; CRT-D, cardiac resynchronisation therapy—defibrillator; IV, intravenous; COPD, chronic obstructive pulmonary disease; CPAP, continuous positive airway pressure; BiPAP, bilevel positive airway pressure; BP, blood pressure; HR, heart rate; ACE, angiotensin-converting enzyme; ARB, aldosterone receptor blocker.

**Table 2:** Factors associated with referral to cardiac rehabilitation at time of discharge among all patients with HF.

Factor	Odds Ratio	95% CI	*p*-Value
Age (years)	0.98	0.96, 0.99	0.001
Gender (female)	0.65	0.42, 1.02	0.06
COPD	0.52	0.31, 0.87	0.01
Anaemia	0.59	0.36, 0.99	0.04
Ischaemic aetiology	1.91	1.27, 2.90	0.01
Rhythm-related aetiology	1.90	1.13, 3.18	0.02
Inpatient oxygen therapy	0.63	0.42, 0.94	0.02
Inpatient PCI	3.31	1.04, 10.48	0.04
Discharge medication: anticoagulant	1.55	1.03, 2.33	0.04

COPD, chronic obstructive pulmonary disease; PCI, percutaneous coronary intervention.

**Table 3:** Factors associated with referral to cardiac rehabilitation at time of discharge, in patients with HFrEF and HFpEF.

Factor	Odds Ratio	95% CI	*p*-Value
HFrEF			
Age (years)	0.99	0.97, 1.00	0.13
Gender (female)	0.47	0.21, 1.04	0.06
Discharge HR	1.03	1.01, 1.05	0.01
Inpatient PCI	4.91	1.27, 18.92	0.02
Inpatient ICD	3.89	1.15, 13.14	0.03
Inpatient CRT-D	3.03	0.92, 9.95	0.07
Discharge medication: antiplatelets	0.46	0.25, 0.85	0.01
HFpEF			
Age (years)	1.02	0.97, 1.07	0.52
Gender (female)	0.58	0.25, 1.36	0.21
Ischaemic aetiology	3.01	1.29, 7.02	0.01
Rhythm-related aetiology	3.08	1.26, 7.53	0.01
Inpatient oxygen therapy	0.39	0.17, 0.88	0.02

HFrEF, heart failure with reduced ejection fraction; HFpEF, heart failure with preserved ejection fraction; HR, heart rate; PCI, percutaneous coronary intervention; ICD, implantable cardioverter defibrillator; CRT-D, cardiac resynchronisation therapy—defibrillator.

## Data Availability

Restrictions apply to the availability of these data. Data was obtained from The Victorian Cardiac Outcomes Register. Applications for permission to access data can be made here: https://vcor.org.au/Data-Access (accessed on 13 February 2022).

## Supplementary Materials

The following are available online at https://www.mdpi.com/article/10.3390/jcm11051232/s1: Table S1. Baseline patient characteristics among all patients and those referred and not referred to cardiac rehabilitation (all data reported).
