# Supplementary material for: Predictors of Referral to Cardiac Rehabilitation in Patients following Hospitalisation with Heart Failure: A Multivariate Regression Analysis
Source: J Clin Med. 2022 Feb 24;11(5):1232. doi: 10.3390/jcm11051232 (PMC8910897; doi:10.3390/jcm11051232)
Supplement: Supplementary file 1 [file jcm-11-01232-s001.zip › jcm-1607804-supplementary.pdf]

Supplementary Table S1: Baseline patient characteristics among all patients and those referred and not referred to cardiac rehabilitation (all data reported)

Characteristics	All (%) (n = 1281)	CR Referral (%) (n = 125)	No CR Referral (%) (n=1156)	*p*-value
Age, y	79.7 (70.3-86.3)	73.6 (62.7-81.5)	80.2 (71.1-86.5)	<0.001
Male, n (%)	723 (56.4)	90 (72.0)	633 (54.8)	< 0.001
BMI, kg/m^2	29.1 (35.8-34.5)	29.7 (25.8-34.9)	29.0 (24.8-34.5)	
**HF Subtype**				
HFrEF	420 (32.8)	62 (49.6)	358 (31.0)	<0.001
HFmrEF	169 (13.2)	11 (8.8)	158 (13.7)	
HFpEF	434 (33.9)	26 (20.8)	408 (35.3)	
Unknown	258 (20.1)	26 (20.8)	232 (20.1)	
LVEF (%)	38.0 (25.6-50.3)	30.0 (22.3-39.8)	40 (26.0-53.0)	0.004
**NYHA**				
Class I / II	20 (4.5) / 236 (53.5)	2 (0.5) /37 (68.8)	18 (4.7) / 198 (51.4)	
Class III/ IV	162 (36.7) / 23 (5.2)	14 (25.9) / 1 (1.9)	148 (38.2) / 22 (5.7)	
Unknown	840 (65.6)	71 (56.8)	770 (66.7)	
**Admission Speciality, n (%)**				0.001
HF Unit	126 (9.8)	18 (14.5)	108 (9.3)	
Cardiology	434 (33.9)	58 (46.8)	376 (32.5)	
Gerontology	36 (2.8)	5 (4.0)	31 (2.7)	
General Medicine	622 (48.6)	39 (31.5)	583 (50.4)	
Other	62 (4.8)	4 (3.2)	58 (5.0)	
**Cardiovascular History, n (%)**				
History of HF	968 (75.5)	93 (74)	874 (75.6)	
Previous hospitalisation for HF	774 (60.4)	75 (60.0)	698 (60.4)	
Cerebrovascular disease	242 (18.9)	27 (21.6)	215 (18.6)	
Hypertension	977 (76.2)	85 (68.0)	891 (77.1)	0.02
History of angina	481 (37.5)	43 (34.4)	437 (37.8)	
History of PCI or CABG	393 (30.7)	37 (29.6)	356 (30.8)	
History of MI	394 (30.7)	40 (32)	353 (30.5)	
Arrythmia	695 (54.2)	66 (52.8)	628 (54.3)	
CIED therapy	284 (22.2)	29 (23.2)	255 (22.1)	0.004
Smoking Status				
Current smoker	133 (12.4)	18 (16.5)	115 (11.9)	
Ex-smoker	504 (46.8)	51 (46.8)	451 (46.8)	
**Heart failure aetiology**				
Ischaemic related cardiomyopathy	458 (35.8)	62 (49.6)	396 (34.3)	0.001
Idiopathic Cardiomyopathy	140 (10.9)	20 (16)	120 (10.4)	
Hypertension	223 (17.4)	16 (12.8)	207 (17.9)	

Infiltrative Cardiomyopathy	10 (0.8)	1 (0.8)	9 (0.8)	
Hypertrophic Cardiomyopathy	43 (3.4)	3 (2.4)	40 (3.5)	
Valvular	179 (14.0)	12 (9.6)	137 (14.4)	
Arrhythmia related	187 (14.6)	26 (20.8)	161 (13.9)	0.04
**Non-Cardiovascular Medical History, n (%)**				
Diabetes	552 (43.1)	56 (44.8)	496 (42.9)	
Dementia	100 (7.8)	4 (3.2)	96 (8.3)	0.04
Depression	251 (19.6)	20 (16.0)	231 (20.0)	
Current malignancy	88 (6.9)	7 (5.6)	81 (7.0)	
COPD / Asthma	394 (30.8)	23 (18.4)	371 (32.1)	0.002
Obstructive sleep apnoea	187 (14.6)	17 (13.6)	170 (14.7)	
Chronic kidney disease				0.002
Mild	241 (18.8)	22 (17.6)	217 (19.0)	
Moderate	408 (31.9)	28 (22.4)	380 (32.9)	
Severe	159 (12.4)	10 (8.0)	149 (12.9)	
Liver disease				
Mild	52 (4.1)	4 (3.2)	48 (4.2)	
Moderate or Severe	30 (2.3)	4 (3.2)	26 (2.3)	
Iron deficiency	253 (20)	16 (12.9)	237 (20.8)	0.04
Anaemia	394 (30.8)	23 (18.4)	371 (32.1)	0.002
**Treatments received during admission, n (%)**				
IV diuretics	1096 (85.8)	102 (82.9)	994 (86.1)	
IV GTN infusion	48 (3.8)	7 (5.7)	41 (3.5)	
IV inotrope infusion	62 (4.8)	10 (8.1)	52 (4.5)	
Oral Diuretics	1161 (90.9)	108 (88.5)	1053 (91.2)	
Oxygen therapy	837 (65.6)	65 (53.3)	772 (66.9)	0.003
CPAP / BiPAP	174 (13.6)	13 (10.6)	161 (14.0)	
IABP/ ECMO	6 (0.5)	1 (0.8)	5 (0.4)	
Invasive mechanical ventilation	25 (2.0)	5 (4.0)	20 (1.7)	
Angiography	112 (8.8)	19 (15.3)	93 (8.1)	0.01
PCI	15 (1.2)	5 (4.0)	10 (0.9)	0.002
CABG	9 (0.7)	1 (0.8)	8 (0.7)	
Dialysis	12 (0.9)	1 (0.8)	11 (1.0)	
LVAD	1 (0.1)	1 (0.8)	00 (0.0)	
Valve procedure	7 (0.5)	1 (0.8)	6 (0.5)	
CIED Therapy				0.002
Pacemaker	28 (2.2)	3 (2.4)	25 (2.2)	
CRT-P	2 (0.2)	0 (0.0)	2 (0.2)	
ICD	15 (1.2)	5 (4.1)	10 (0.9)	
CRT-D	18 (1.4)	13 (1.1)	5 (4.1)	
**Resting Haemodynamics on d/c**				
Systolic BP (mmHg)	120 (110.0-135)	118 (110-130)	120 (110-135)	0.02
Diastolic BP (mmHg)	68 (60-75)	68 (60-75)	68 (60-75)	
HR (bpm)	74.0 (65-83)	75.0 (65-85)	74 (65-82)	
**Heart Failure pharmacotherapy,**				

**n (%)**				
ACE Inhibitor	534 (41.8)	54 (43.2)	480 (41.6)	
ARB	219 (17.1)	24 (19.4)	195 (16.9)	
Beta blocker	910 (71.2)	98 (79.0)	812 (70.4)	0.04
Aldosterone antagonist	474 (37.1)	48 (38.7)	426 (36.9)	
Digitalis	210 (16.4)	15 (12.1)	195 (16.9)	
Antiarrhythmic	141 (11.0)	18 (14.5)	123 (10.7)	
Nitrate	217 (17.0)	17 (13.7)	200 (17.3)	
Loop diuretic	1207 (94.2)	118 (94.4)	1089 (94.2)	
Other vasodilator	62 (4.9)	3 (2.4)	59 (5.1)	
Ivabradine	45 (3.5)	6 (4.8)	39 (3.4)	
Lipid lowering agent	715 (55.9)	73 (58.4)	642 (55.6)	
Antiplatelet	674 (52.7)	60 (48.0)	614 (53.2)	
Anticoagulant	586 (45.8)	69 (55.6)	517 (44.8)	0.02
Thiazide diuretic	140 (11.0)	11 (8.9)	129 (11.2)	
Calcium channel antagonist	209 (16.4)	21 (16.9)	188 (16.3)	
Total Meds on d/c (SEM)	10.0 (8.0-13)	9 (8-11)	10 (8-13)	0.047

Data expressed as median and percentiles (25-75%) for continuous variables and count and proportions (%) for categorical variables.

ǂ Variables where missing data >15%.
ɫwhere factor has more than one level, p-value applies to the overall association of this factor with the outcome.

BMI, body mass index; HFrEF, heart failure with reduced ejection fraction; HFmrEF, heart failure with mid-range ejection fraction; HFpEF, heart failure with preserved ejection fraction; LVEF, left ventricular ejection fraction; HF, heart failure; PCI, percutaneous coronary intervention; CABG, coronary artery bypass graft; MI, myocardial infarction; CIED, Cardiac Implantable Electronic Device; COPD, chronic obstructive pulmonary disease; IV, intravenous; GTN, Glyceryl trinitrate; Cardiac Implantable Electronic Device; CPAP, continuous positive airway pressure; BiPAP, bilevel positive airway pressure, IABP, intra-aortic balloon pump; ECMO, extracorporeal membrane oxygenation; LVAD, left ventricular assist device; CRT-p, cardiac resynchronisation therapy – pacemaker; ICD, implantable cardioverter defibrillator; CRT-D, cardiac resynchronisation therapy – defibrillator; BP, blood pressure; HR, heart rate; ACE, angiotensin converting enzyme; ARB, aldosterone receptor blocker
